# Supplementary material for: Patterns of Intron Gain and Loss in Fungi
Source: PLoS Biol. 2004 Nov 30;2(12):e422. doi: 10.1371/journal.pbio.0020422 (PMC532390; doi:10.1371/journal.pbio.0020422)
Supplement: Table S1 — Also available at http://genes.mit.edu/NielsenEtAl/. (4.3 MB ZIP). [file pbio.0020422.st001.zip › NielsenEtAl/html/264.html]

AN1673.1.NCU02785.1.MG08596.1.FG01486.1


```
 CLUSTAL W (1.82) Multiple Sequence Alignments - Introns Inserted


Sequence 1: NCU02785.1	481 aa
Sequence 2: MG08596.1	466 aa
Sequence 3: FG01486.1	469 aa
Sequence 4: AN1673.1	482 aa
Alignment Length: 495 aa
Number Identitical Residues: 243 aa
Alignment Score (without introns) 11698


MG08596.1 	----MTDTTQGWAPDS~WRSKPIKQCPEYPDQEALKKATTQLKRLPPIVHPRE~IERLKE
NCU02785.1	MSQQTTPNAPGWAPDS~WRSKPIKQCPEYPDKAALEKATNELKTLPPIVLPNE~IIRLRE
FG01486.1 	---MASSSSAAWSPSS~WRSKPMKQSPNYPDPEPLAKAVQELSDLPPLVHPNE~IIALKA
AN1673.1  	------MGAEDWNPKS1WTSKPIKQDVVYEDVEGVQSALRKLEKLPPLVTTHE0IANLKK
          	        :  * *.* * ***:**   * *   : .*  :*. ***:* ..* *  *: 

MG08596.1 	LLSDVAHGKAFLLQGG~DCAELFDYCEQSTIESKIKLLLQMSLVLIWG~GNKRVVRIGRM
NCU02785.1	HLRDVAQGKAFLLQGG~DCAELFSYCQQDVIESKIKLLLQMSLVLLWG~ADKPVVRIGRM
FG01486.1 	HLRDVAQGNAFLLQGG~DCAELFDYCRQGPIESKIKLLLQMSVVLIWG~TNKRVVRIGRM
AN1673.1  	SLKNVALGKAFVLQGG1DCAELFDYCNQDMIEAKVKLLLQMSLVLIWG1ANMPVVRIARI
          	 * :** *:**:**** ******.**.*. **:*:*******:**:**  :  ****.*:

MG08596.1 	AGQYAK~PRSSPTEIVDGREVPSFRGDILNGFPL--DQRELDPGRMVQAYHHSSATLNYV
NCU02785.1	AGQYAK~PRSSPVETINGKEVPSFRGDILNGFHP--DERELDPNRLVRAYQYSSATLNYI
FG01486.1 	AGQYAK~PRSSPTEMVDGKEVPSFKGDIINGFRL--EDREIDPNRLVKAYHHSAATLNYI
AN1673.1  	AGQFAK2PRSNPMEVVNGVEMPSFRGDNINGFDATPDSRRPDPSRLVSAYFHSAATLNYL
          	***:** ***.* * ::* *:***:** :***  :.:.*. **.*:* ** :*:*****:

MG08596.1 	RASISSGIADLHRPLDWGLGHVRDATLKAKYEQAVDFLTESLRFMQTIGADKSPNLGTVE
NCU02785.1	RGAIGSGIADLHGPLDWGLGHVRDPALKSKYQETVDRIQEMLRFMHTIGADQNEKLSTVE
FG01486.1 	RAALASGIADLHRPLDWGLGHVRDPELKEKYSAIASSIQQTLRFLQVINARPGE-LDSVE
AN1673.1  	RASLSSGLADLHSPLDWGLGHVITPSIKEKYERIVNRVKDALRFMQTVGIDTDRGVETVD
          	*.::.**:**** *********  . :* **.  .. : : ***::.:.   .  : :*:

MG08596.1 	LYTSHEGLLLEYEEPLTRLLENPSTATKGDEV------------TAG--KKYYDTSAHFL
NCU02785.1	LFTSHEGLLLEYEEPLTRLLNHPSVRSYPPDS------------TTPPKKEYYNTSAHFL
FG01486.1 	LFTSHEGLLLDYEQPLTRLLDKPTTRTVSPTPP----------GSDGEKKEYYDTSAHFI
AN1673.1  	IYTSHEGLLLEYETSLTRLLKDPTPPSSHQPTVTLVPGKGPIPAQTPASSSYYATSSHFL
          	::********:** .*****..*:  :      :  ..... ..    ...** **:**:

MG08596.1 	WIGDRTRQIDGAHVEFFRGIANPIGVKVGPSTPASDLLSLLRKLNPDREAGKITLITRYG
NCU02785.1	WIGDRTRQIDHAHVEYFRGIANPIGVKIGPSTPTSDLLPMLRTLNPNREPGKVTLITRYG
FG01486.1 	WIGDRTRQIDHAHVEFFRGIANPIGIKVGPTTPASDLLSLLRTLNPSCEPGKITLITRYG
AN1673.1  	WIGDRTRQLTGAHVEFFRGIANPIGIKIGPSMTPTELVTLLDTVNPTREIGKVTLISRYG
          	********:  ****:*********:*:**: ..::*:.:* .:**  * **:***:***

MG08596.1 	ADRVAELLPAHIRAVEDSEYRGLAVWQCDPMHGNTRSVG-NFKTREFKDIFEELSQTLRI
NCU02785.1	ADKVASLLPAHIRTVESSEYARTVVWQCDPMHGNTQSVSGGIKTRKFSDIFSELQQTLRI
FG01486.1 	AAKVGDLLPTHIRAVEDSEYRRTVVWQCDPMHGNTLSTPTGIKTRRFGDIYRELQETLRI
AN1673.1  	AANIAAHLPGHISAVQSS--GHIPVWQCDPMHGNTRSTPNGVKTRHFSDILSELKQALEI
          	* .:.  ** ** :*:.*      *********** *.  ..***.* **  **.::*.*

MG08596.1 	HREEGSYLGGVHLELTGDAVTECLGGAAELEASDLPTNYTTFCDPRLNEKQALELAFLVA
NCU02785.1	HKEEKSYLGGMHLELTGDAVTECLGGGAGLDEDDLSTNYTSFCDPRLNEKQALELAFLVA
FG01486.1 	HKEQGSNLGGMHLELTGDAVTECLGGSEDLGEDDLSTNYTSFCDPRLNEKQALELAFLVA
AN1673.1  	HKAAGSFLGGMHLELTGEAVTECVGGAGGLTEEGLGERYTTFCDPRLNEKQALELAFLVA
          	*:   * ***:******:*****:**.  *  ..*  .**:*******************

MG08596.1 	DHYRKGRTPPTC--------
NCU02785.1	DHYRQERKEKEAERRKSSVV
FG01486.1 	DHFSREEKAV----------
AN1673.1  	GFYREMEGGEGVNSI-----
          	..: . .     .
```
